# Estimating family planning coverage from contraceptive prevalence using national household surveys

**DOI:** 10.3402/gha.v8.29735

**Published:** 2015-11-09

**Authors:** Aluisio J. D. Barros, Ties Boerma, Ahmad R. Hosseinpoor, María C. Restrepo-Méndez, Kerry L. M. Wong, Cesar G. Victora

**Affiliations:** 1 International Center for Equity in Health, Federal University of Pelotas, Pelotas, Brazil; 2 World Health Organization, Geneva, Switzerland

**Keywords:** family planning, contraception, coverage, prevalence, health indicators

## Abstract

**Background:**

Contraception is one of the most important health interventions currently available and yet, many women and couples still do not have reliable access to modern contraceptives. The best indicator for monitoring family planning is the proportion of women using contraception among those who need it. This indicator is frequently called *demand for family planning satisfied* and we argue that it should be called *family planning coverage* (FPC). This indicator is complex to calculate and requires a considerable number of questions to be included in a household survey.

**Objectives:**

We propose a model that can predict FPC from a much simpler indicator – contraceptive use prevalence – for situations where it cannot be derived directly.

**Design:**

Using 197 Multiple Indicator Cluster Surveys and Demographic and Health Surveys from 82 countries, we explored least-squares regression models that could be used to predict FPC. Non-linearity was expected in this situation and we used a fractional polynomial approach to find the best fitting model. We also explored the effect of calendar time and of wealth on the models explored.

**Results:**

Given the high correlation between the variables involved in FPC, we managed to derive a relatively simple model that depends only on contraceptive use prevalence but explains 95% of the variability of the outcome, with high precision for the estimated regression line. We also show that the relationship between the two variables has not changed with time. A concordance analysis showed agreement between observed and fitted results within a range of ±9 percentage points.

**Conclusions:**

We show that it is possible to obtain fairly good estimates of FPC using only contraceptive prevalence as a predictor, a strategy that is useful in situations where it is not possible to estimate FPC directly.

**Paper context**

Measuring progress towards universal health coverage involves producing estimates of health intervention coverage. For that purpose, coverage indicators that are accurate and easy to collect are needed. The existing indicator to estimate contraceptive use among women who need it (family planning coverage, or FPC) is complicated because it depends on several items of information that are difficult to ascertain. We propose, for situations where this complex indicator is not available, a way to estimate it from a much simpler indicator, namely the proportion of women using contraception in the population. This estimate is precise enough to be used as a substitute for FPC where it is not possible to estimate the latter directly.

Family planning is one of the most important health interventions currently available, and the global reduction in family size has contributed substantially to decreases in maternal and under-five mortality (1, 2). One review, including a simulation of the impact of different levels of investment in health, concluded that expanding access to modern contraceptives was the most cost-effective intervention, with great potential for reducing child and maternal deaths (3). It has also been argued that important social and economic benefits of increased access to family planning, such as improved women's earnings and more investments in schools, are expected. Economic growth was also linked to reduction in fertility rates because of increased participation of women in the labour force and reduction of young dependents in the households (4). Yet many women and couples still do not have reliable access to modern contraceptives. Recently, a global partnership, Family Planning 2020 (5), was established to support the rights of women and girls to decide, freely and for themselves, whether, when, and how many children they want to have.

The monitoring of progress towards the Millennium Development Goal target for achieving universal access to reproductive health (also known as *Target 5B*) involves two indicators related to contraception: contraceptive use prevalence [contraceptive prevalence rate (CPR)] and unmet need for family planning. The Commission on Information and Accountability for Women's and Children's Health proposed an alternative indicator, percent of demand for family planning satisfied (FPS) (6).

In addition to demographic and health impact, progress monitoring should be based on intervention coverage indicators, which are defined as the proportion of people receiving the services they need. This is justified because coverage indicators respond more promptly to programme changes than impact indicators, thus representing an invaluable tool for programme evaluation and consequent correction (7). CPR is a simple current status indicator to compute and understand: the percentage of women (15–49 years of age, currently married or in union) who are using any type of contraception. It is, however, not a true coverage indicator as not all women are in need of contraceptives, making it difficult to set meaningful targets. Unmet need indicators are more complex and have been frequently misunderstood (8), both in terms of their interpretation and in terms of their mathematical interrelationships. There is especially a tendency to suppose that percentage met need is 100 minus unmet need, which is not true given that the denominator of both indicators include women who do not need contraception. Recently, a target of 75% for an FPC indicator – percent of demand satisfied – has been proposed in the context of measuring progress towards the goals of family planning for 2020 and beyond (9).

In this paper, we first argue that family planning demand satisfied is the most useful indicator of coverage and propose that it should be referred to as *FPC*. The data requirements for the FPC indicator are quite considerable and it is less commonly measured in surveys than CPR, hampering comparisons between populations, equity analyses, and trend assessments. Therefore, our analysis aims to show how data on CPR can be used to estimate the FPC indicator in different settings.

## Methods

### Indicators

**Fig. 1:**
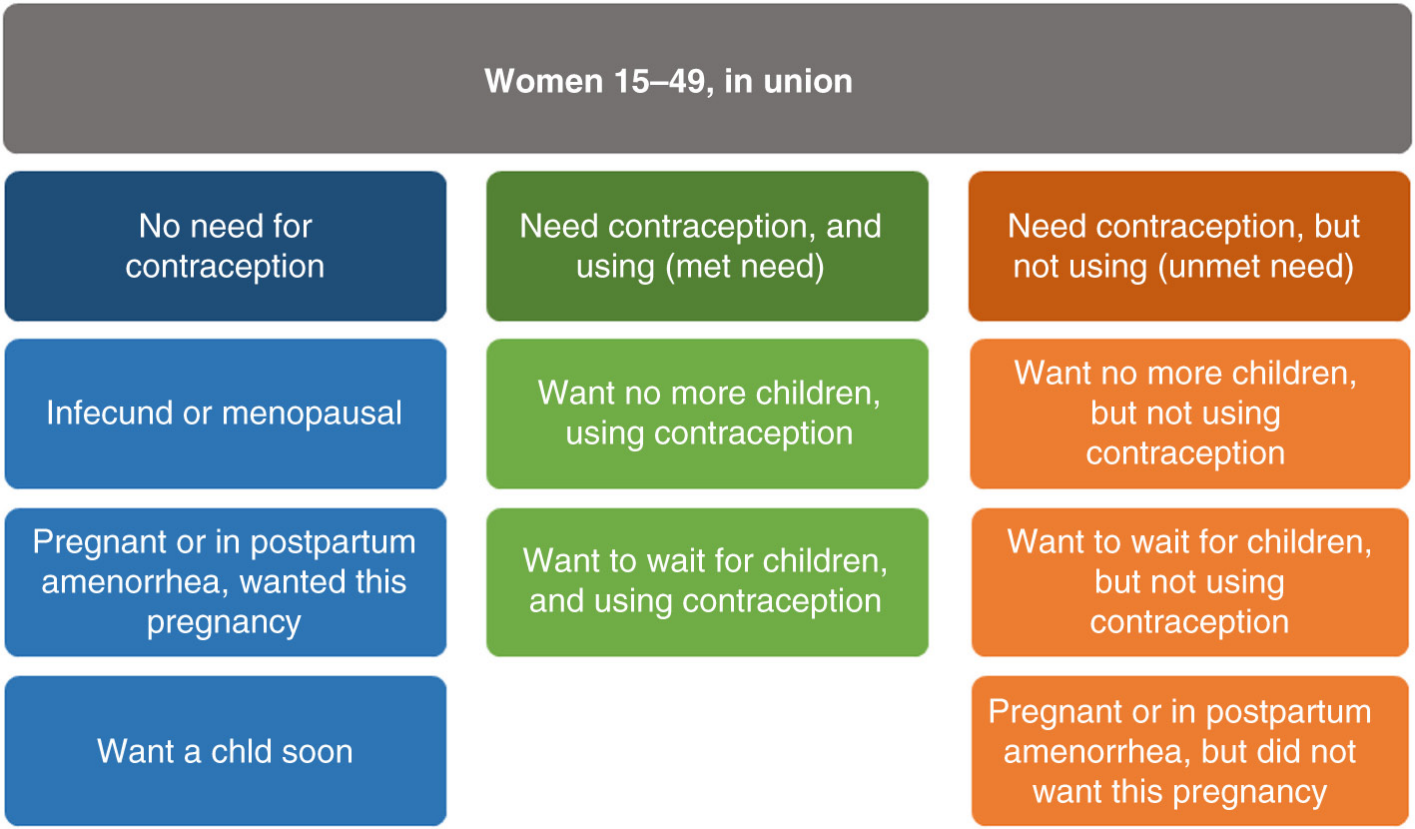
Groups of women in terms of need for contraception and its use.

The calculation of either unmet need or FPC requires dividing women of fertile age into groups of need for contraception. The first group includes women who are infecund or menopausal, women who are pregnant or in postpartum amenorrhoea who wanted the pregnancy, and women who want another child in the next 2 years (Fig. 1, groups in blue). In this group, we have women with no need for contraception. The second group includes women who do need contraception, because they want no more children, want to wait, or did not want the current or recent pregnancy. These women can be further divided into those using contraceptives (Fig. 1, green), and those not using contraceptives (Fig. 1, orange). Unmet need for family planning is defined as the proportion of women with unmet need in relation to all women [orange/(blue+green+orange)]. This is probably the most common indicator used in the literature, but it is important to note that the denominator includes women who do not need contraception and thus, like CPR, it is not a coverage indicator.

FPC is a ‘positive’ (in the sense that higher is better) alternative to unmet need and the only true coverage indicator, being defined as the proportion of women that need contraception who are actually using contraceptives [green/(green+orange)]. As with all coverage indicators, it is easily interpretable in the sense that it can actually vary from 0 to 100%. From here on, we will refer to this indicator as *FPC*.

Given that the denominators of these two indicators are different, it is easy to see that FPC is not one minus unmet need, as is sometimes commonly believed.

All current definitions refer to women who are married or in union. It is, however, possible to simply extend the computations to include all sexually active women, including those who are not married or in a union. Another alternative to the standard survey definition is to estimate CPR and FPC only for modern contraceptives.

Details for the definitions of women that are infecund or in postpartum amenorrhoea are presented in a revision for unmet need definition published by the Demographic and Health Survey (DHS) programme (10). Despite the simplification proposed in the definitions of *unmet need* and *FPC*, the classification of women into the groups needed to estimate the indicators is complex and requires the inclusion of a large number of questions in a survey, some of them very subjective. Following Fig. 1, FPC (the proportion of women using contraception among those who need it) and CPR (the proportion of women using contraception) can be written as

$$\text{FPC}=\frac{\text{women using contraception}(\text{green})}{\text{women in need of contraception}(\text{green}+\text{orange})}$$

$$\text{CPR}=\frac{\text{women using contraception}(\text{green})}{\text{all women}(\text{blue}+\text{green}+\text{orange})}$$

Dividing both the numerator and denominator of FPC by the total number of women aged 15–49 years (blue+green+orange) we get

FPC=CPR/%women in need of contraception(green+orange)

and it therefore follows that

FPC=CPR/(1-% women not in need of contraception (blue))

Therefore, FPC depends on CPR and the proportion of women not in need of contraception.

Many surveys do not include the full array of questions required to obtain a direct estimate of unmet need or FPC. An estimate of coverage may however be needed, given the importance of FPC when not only CPR changes but also the desire for children, the need to assess trends over time using different surveys, or the use of FPC in combined health intervention coverage indicators such as the composite coverage indicator (11). The strong correlation between FPC and CPR has already been recognised in the literature (12) and used to predict FPC in surveys where only CPR was available.

In this work, we take this approach further exploring the relationship between FPC and its defining variables (CPR, proportion of women currently wanting children, proportion of women pregnant or in postpartum amenorrhea, and proportion of infecund or menopausal women) and proposing a predictive model for FPC.

### Data and analytical methods

We used DHS and Multiple Indicator Cluster Surveys (MICS) undertaken since the 1990s to explore the relationship between FPC and CPR and determine a predictive model that may be used as a simpler method to estimate FPC where it is not possible to estimate it directly. It may be useful in small surveys where there is interest in FPC but the number of questions involved is too large or for some DHS and MICS surveys where not all relevant questions were included. It may be also relevant when we want to estimate the composite coverage index, an indicator that involves an estimate for FPC along with other seven coverage indicators (13).

From a set of 238 DHS and MICS surveys, FPC was available for 173 DHS and for 24 MICS, totalling 197 surveys used for the modelling exercise, from 82 countries. Survey years ranged from 1993 to 2012. A full list of countries and respective surveys is presented in the Supplementary file.

All reproductive estimates used in this analysis refer to women aged 15–49 years who were married or in union at the time of the survey. CPR was defined as the prevalence of contraceptive use, either modern or traditional.

A revised definition of FPC was presented in 2012 (10) by a working group whose main objective was to make the indicator simpler and dependent upon fewer survey questions. In our analysis, however, we used the pre-2012 definition, because this was the one readily available from most surveys. In numerical terms, the difference between the two definitions was very small for the 21 surveys where both were available. The average difference between old and new definitions was 1.3 percentage points (95% limits of agreement –1.1 to 3.7 percentage points). A similar result was found for unmet need, with an average difference of 1.7 percentage points (10).

We explored the Pearson correlations between FPC, CPR, and the proportion of women wanting/having more children or who were infecund or menopausal. We also calculated the semi-partial correlations between these variables to estimate the percentage of CPR variability explained by both predictors after controlling for the others. Modelling was done using linear regression on a logit transform of FPC to avoid predicted values outside the [0–1] interval. We explored a range of models, using a fractional polynomial approach (14), in order to find the best performing one. In such models, we corrected the standard errors by taking into account repeated surveys for a particular country as a cluster. We also explored the relationship between FPC and CPR using estimates stratified by wealth quintiles. Similar analyses were performed and standard errors corrected by clusters of surveys.

The predicted values for FPC were back-transformed to its original scale in order to make results easier to understand.

## Results

Table 1 shows the distribution of our study variables: FPC, CPR, and the proportion of women wanting/having more children or who were infecund or menopausal. Both FPC and CPR varied widely – FPC from 12 to 94% and CPR from 3 to 79%. The percentage of women wanting/having more children varied from 4 to 53%, and the percentage of infecund or menopausal women varied from 5 to 37%.

**Table 1:** Mean, minimum, maximum, median, and 10th and 90th percentiles of the percentages of family planning coverage (FPC), contraceptive prevalence (CPR), women who want more children, and women who are infecund or menopausal

Variable	Mean	Minimum	10th percentile	Median	90th percentile	Maximum
FPC	61.7	11.8	30.0	62.8	89.3	94.3
CPR	40.6	2.8	12.6	40.0	70.5	79.0
% wanting/having more children	17.7	3.8	5.0	15.6	34.2	52.5
% infecund or menopausal	13.0	4.5	7.2	12.6	18.4	37.0

Source: DHS and MICS: 197 surveys from 1993 to 2012.

The correlations between FPC and its potential predictors (CPR, percentage of women wanting/having more children, and proportion of infecund or menopausal women) were 0.97, −0.76, and −0.61, respectively. We also observed strong negative correlation between CPR and the percentages of women wanting/having more children and those infecund or menopausal (−0.84 and −0.69, respectively).

The semi-partial squared correlations for percentages of women wanting/having more children and those infecund or menopausal adjusting for each other and for CPR were 0.8 and 0.6 percentage points, respectively, indicating that the addition of both variables in a predictive model where CPR was already included would add less than 1.5 percent points in its R^2^. This is explained by the strong correlation that exists between these predictors and CPR. Therefore,it is clear that we can model FPC using only CPR as a predictor, since the addition of the other predictors would improve any model only marginally.

Using the fractional polynomial strategy to find the best way to fit CPR in a model to predict the logit of FPC resulted in a model where CPR appears twice with powers one and two. This model can be written as follows:

$$\text{logit}(\text{FPC})=0\text{.61}+\text{0.68 log}(\text{CPR})+\text{3.57 CP}{\text{R}}^{\text{2}}$$

**Fig. 2:**
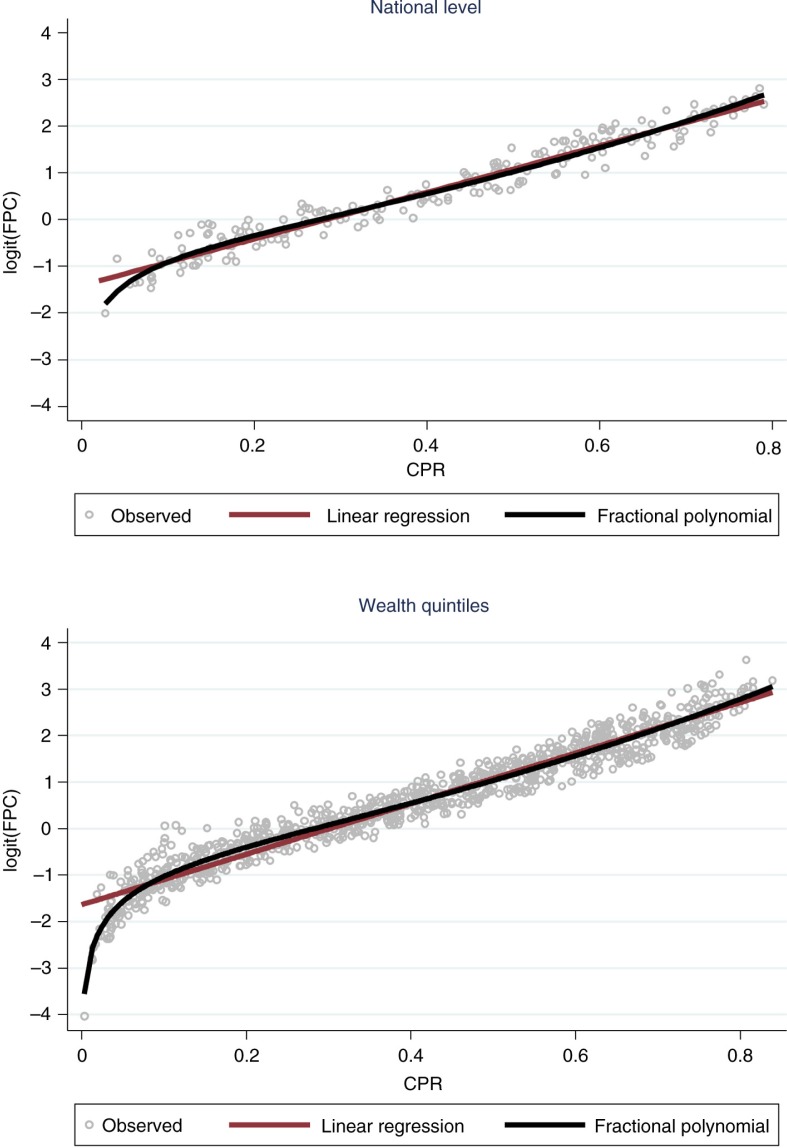
Scatter plots plus linear and fractional polynomial regressions at national level and by wealth quintiles, showing the relationship between logit family planning coverage (FPC) and contraceptive prevalence rate (CPR). Source: DHS and MICS: 197 surveys from 1993 to 2012.

Compared to the simpler linear model, this alternative is better both in terms of reducing the deviance (*p=*0.028) and visually (Fig. 2). The results suggest that the association between the logit of FPC and CPR is not linear, especially when CPR is low. The proposed model explains 94.7% of the FPC variation (Table 2).

**Table 2:** Fractional polynomial model for predicting family planning coverage (FPC) from contraceptive prevalence rate (CPR)

Variable	Coefficient	*p*	95% CI			
National level						
Intercept	0.61	<0.001	0.38	0.84	*N=*197	*R* ^2^=94.7%
Log(CPR)	0.68	<0.001	0.55	0.80		
CPR^2^	3.57	<0.001	3.15	3.99		
Wealth quintiles						
Intercept	0.66	<0.001	0.51	0.80	*N=*959	*R* ^2^=95.5%
Log(CPR)	0.75	<0.001	0.67	0.82		
CPR^2^	3.58	<0.001	3.27	3.89		

Source: DHS and MICS, 197 surveys from 1993 to 2012.

Our next step was to assess whether this relationship changed over time. For that, we added the year of the survey to the previous model and found no effect (*p*=0.348). We also tested the interaction between year and CPR and again found no effect (*p*=0.328).

Finally, we explored whether wealth was an effect modifier of the association between FPC and CPR. For that, we used estimates of FPC and CPR stratified by wealth quintiles, from the same surveys described above. Figure 2 also shows the linear and fractional polynomial models for the data stratified by wealth quintiles, prior to adjustment by wealth. The results obtained are very similar to those at the national level (Table 2), and now with more data points at the low end of the CPR it is clear that the inflection of the curve fits the data well.

We found that there is an independent effect of wealth quintiles (*p*<0.001), but not effect modification (*p*=0.375). The results show that, for the same level of CPR, FPC increases slightly with wealth. The maximum difference between the poorest and the richest quintiles was observed when CPR was around 30%. At this point, FPC for the richest was only three percentage points higher than for the poorest (see Fig. A1 in the Supplementary file).

**Fig. 3:**
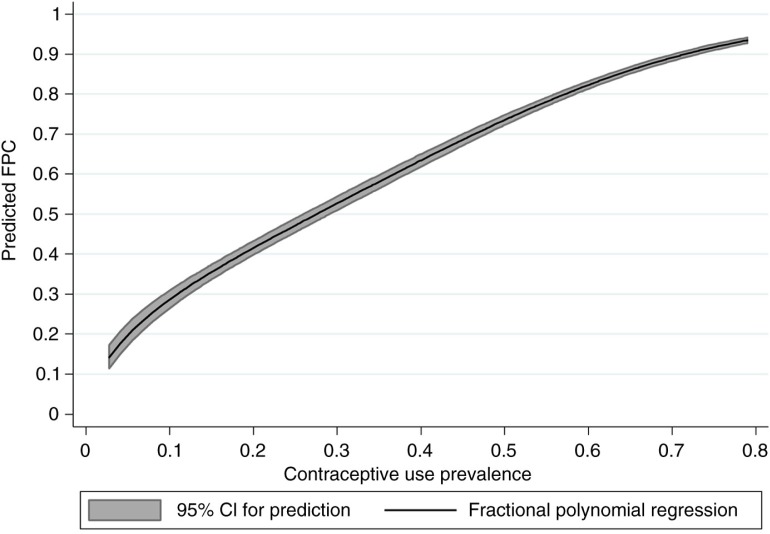
Predictive model for family planning coverage (FPC) based on contraceptive prevalence rate.

Given that we found no important effect of either time or wealth, our predictive model of FPC using CPR as the sole predictor is warranted. The analysis of the residuals suggested that the fit is good (details in the Supplementary file). We show in Fig. 3 the prediction curve with 95% confidence interval for the predicted average. A concordance analysis between predicted and observed values of FPC showed that the 95% limits of agreements were ±9.3 percentage points (see Fig. A4 in the Supplementary file).

We also present, in Table 3, predicted values for FPC at selected levels of CPR. The predictions in the original FPC scale were obtained by back-transforming the results in the logit scale.

**Table 3:** Predicted values of family planning coverage (FPC) for a series of contraceptive prevalence (CPR) levels

CPR coverage (%)	Estimated FPC (%)	CPR coverage (%)	Estimated FPC (%)	CPR coverage (%)	Estimated FPC (%)
1^a^	8^a^	35	58	70	89
5	20	40	64	75	92
10	29	45	69	80	94
15	36	50	74	85^a^	96^a^
20	42	55	78	90^a^	97^a^
25	47	60	82	95^a^	98^a^
30	53	65	86	99^a^	98^a^

a Predictions outside the data range for CPR (2–79%).

## Discussion

FPC can be computed from surveys and is the most suitable indicator for the monitoring of progress of family planning programmes. However, the estimation of FPC involves a large number of questions in a survey and it is rather complex to calculate. The 2012 revised definition of *unmet need* tried to simplify its calculation and make it less dependent of unreliable questions (10). Still, around 15 questions are needed to establish all the defining items of this indicator, including whether 1) the woman is pregnant; 2) the woman is fecund; 3) she wants another baby within 2 years; 4) she is using contraception. A full list of the questions used in a DHS survey is presented in the DHS report ‘Revising unmet need for family planning’ (Appendix A in Ref. 10). CPR is a much simpler indicator and involves just asking whether the woman is using a contraceptive and, if yes, which one. However, it has several limitations. Most importantly, it is not a coverage indicator, as it will not (and should not) reach 100%, and there is no clear indication as to what is the desired prevalence for a given country.

Our analysis shows that it is possible to obtain a reliable and precise estimate of FPC from CPR alone. Using a large number of surveys covering a wide period (1993–2012) and different statistical models, we found a very strong association between FPC and CPR, yielding a predictive model that could explain 95% of the FPC variability. We also used estimates by wealth quintile to check whether the observed association at the country level would be different for wealth groups. The approach used to fit the models, fractional polynomials, was selected in order to allow for non-linearity in the association, while offering a large family of curves to choose from (14).

At a first look, being able to predict FPC from CPR alone may be a surprise given all the complexity involved in the definitions of FPC and unmet need, which include asserting women's current status in relation to fecundity, pregnancy, desire for more children, and so on. The answer lies in the empirical evidence that the defining variables of FPC, as we have shown, are highly correlated. Therefore, it is possible to make precise estimates of FPC without the need to take into account the other variables used in the calculation of FPC.

The increased sample size and variability of CPR offered by the analysis by wealth quintiles strengthened the results found at the country level. Specifically, it was possible to assess in more detail the shape of the association when CPR was very low, below 10%, which was more common for the poorest groups. It was also possible to show that there was no effect modification in the association between FPC and CPR by wealth group. The implication is that there is no need for adjustment for wealth or for group-specific predictions.

As expected, the proportion of women who want a child within 2 years is associated with FPC at the country level, but it makes very little difference to the final predictive model and can be ignored for all practical purposes.

The FPC indicator, as well as the conventional CPR, is limited to women who are married or in union. This is a drawback, as the use of family planning methods is at least as important for those not living in a union and exposed to the risk of pregnancy as for those in union (9). Many surveys report the proportion of sexually active unmarried women using contraception as well. Technically, a good additional FPC indicator would include all sexually active women of reproductive ages (i.e. exposed to the risk of pregnancy). This shift is beyond the scope of this paper, but the methods used here can be applied in the same way to find the best way to predict FPC among all sexually active women from CPR among all sexually active women at reproductive ages.

It has been proposed to focus the coverage indicator on modern contraceptives only (9). Here, we used both modern and traditional methods, but the method could easily be adapted to estimate coverage with modern contraceptives from the modern CPR.

Household surveys are the predominant source of data on contraceptive prevalence. Health facility data on current users and estimates of the population at risk (married women or sexually active women) could also provide an estimate of the CPR, which could then be converted into a coverage indicator. Currently, little use is made of such approaches because of data quality issues with facility data on family planning, such as incomplete reporting and double counting (see, e.g. www.cpc.unc.edu/measure/prh/rh_indicators/specific/fp/cpr).

As many countries embark on universal health coverage, monitoring progress becomes increasingly important. The WHO/World Bank Universal Health Coverage monitoring framework proposes a focus on a set of indicators of intervention coverage and financial protection (15). FPC is considered a good indicator for all countries and could be used as a tracer indicator (7). The statistical model proposed in this paper thus contributes to comparable monitoring of universal health coverage by improving the availability of data on FPC by socio-economic and other characteristics.

## Conclusions

FPC, also known as *demand for FPS*, is an important indicator for monitoring and policy making. It is also proposed as an indicator for monitoring one of the health targets of the sustainable development goals. In low- and middle-income countries, the FPC indicator is usually estimated from household surveys. However, it requires asking many additional questions, including some regarding exposure to the risk of pregnancy, fecundity, and desire for children. Many surveys only provide data on contraceptive prevalence and do not have adequate information to directly estimate FPC. Thus, we developed a predictive model that is at the same time simple and precise, making it possible to produce a very credible estimate of FPC using only contraceptive prevalence, which is an indicator obtained much more easily from surveys. Given that direct measurement of FPC takes into account several aspects such as whether women are fecund, want more children soon, and so on, along with their use of contraceptives, it may be surprising that we successfully tested such a simple model. The explanation is that all of these variables are highly correlated with each other, making such a model possible. FPC can reliably be predicted from contraceptive prevalence data using a simple mathematical model, which permits analysis of FPC trends and differentials using different types of surveys and allows the use of FPC in, for instance, analysis of progress towards universal health coverage.

## Supplementary Material

Estimating family planning coverage from contraceptive prevalence using national household surveys
