# Corrigendum

**DOI:** 10.3402/gha.v8.30442

**Published:** 2015-12-04

Regarding the paper titled: “Estimating family planning coverage from contraceptive prevalence using national household surveys” by Aluisio J. D. Barros, Ties Boerma, Ahmad R. Hosseinpoor, María C. Restrepo-Méndez, Kerry L. M. Wong, Cesar G. Victor

Published in *Global Health Action* (section “Original Articles”) 9 November 2015. Citation: Glob Health Action 2015, 8: 29735 - http://dx.doi.org/10.3402/gha.v8.29735

The conflict of interest and funding section is incomplete.

This section currently reads:

**Competing interests and funding:** The authors have not received any funding or benefits from industry or elsewhere to conduct this study.

The section should read:

Competing interests and funding: The activities of the International Center for Equity in Health (ICEH) and the authors Aluisio J D Barros and Cesar G Victora are supported by a Investigator Award granted by the Wellcome Trust, UK.

