# Supplementary material for: Estimating family planning coverage from contraceptive prevalence using national household surveys
Source: Glob Health Action. 2015 Nov 9;8:10.3402/gha.v8.29735. doi: 10.3402/gha.v8.29735 (PMC4642361; doi:10.3402/gha.v8.29735)
Supplement: Estimating family planning coverage from contraceptive prevalence using national household surveys [file GHA-8-29735-s001.pdf]

Supplementary File: Estimating family planning coverage from contraceptive prevalence using national household surveys

Aluisio J D Barros, Ties Boerma, Ahmad Reza Hosseinpoor, María Clara Restrepo-Méndez, Kerry Wong, Cesar G Victora. Global Health Action 2015; 8:29735.

Data source for the analyses

S1 Table – List of surveys used in the present analyses.

Order	Country	Year	Source
**1.**	Albania	2008	DHS
**2.**	Armenia	2000	DHS
**3.**	Armenia	2005	DHS
**4.**	Armenia	2010	DHS
**5.**	Azerbaijan	2006	DHS
**6.**	Bangladesh	1993	DHS
**7.**	Bangladesh	1996	DHS
**8.**	Bangladesh	1999	DHS
**9.**	Bangladesh	2004	DHS
**10.**	Bangladesh	2007	DHS
**11.**	Bangladesh	2011	DHS
**12.**	Benin	1996	DHS
**13.**	Benin	2001	DHS
**14.**	Benin	2006	DHS
**15.**	Bolivia	1994	DHS
**16.**	Bolivia	1998	DHS
**17.**	Bolivia	2003	DHS
**18.**	Bolivia	2008	DHS
**19.**	Bosnia and Herzegovina	2006	MICS
**20.**	Brazil	1996	DHS
**21.**	Burkina Faso	1998	DHS
**22.**	Burkina Faso	2003	DHS
**23.**	Burkina Faso	2006	MICS
**24.**	Burkina Faso	2010	DHS
**25.**	Burundi	2010	DHS
**26.**	CAR	1994	DHS
**27.**	CAR	2006	MICS
**28.**	Cambodia	2000	DHS
**29.**	Cambodia	2005	DHS
**30.**	Cambodia	2010	DHS
**31.**	Cameroon	1998	DHS
**32.**	Cameroon	2004	DHS
**33.**	Cameroon	2011	DHS
**34.**	Chad	1996	DHS
**35.**	Chad	2004	DHS
**36.**	Colombia	1995	DHS
**37.**	Colombia	2000	DHS
**38.**	Colombia	2005	DHS
**39.**	Colombia	2010	DHS
**40.**	Comoros	1996	DHS
**41.**	Congo Brazzaville	2005	DHS
**42.**	Congo Brazzaville	2011	DHS
**43.**	Congo DR	2007	DHS
**44.**	Cote d'Ivoire	1994	DHS
**45.**	Cote d'Ivoire	1998	DHS

Order	Country	Year	Source
**46.**	Cote d'Ivoire	2006	MICS
**47.**	Cote d'Ivoire	2011	DHS
**48.**	Cuba	2006	MICS
**49.**	Djibouti	2006	MICS
**50.**	Dominican Republic	1996	DHS
**51.**	Dominican Republic	1999	DHS
**52.**	Dominican Republic	2002	DHS
**53.**	Dominican Republic	2007	DHS
**54.**	Egypt	1995	DHS
**55.**	Egypt	2000	DHS
**56.**	Egypt	2005	DHS
**57.**	Egypt	2008	DHS
**58.**	Ethiopia	2000	DHS
**59.**	Ethiopia	2005	DHS
**60.**	Ethiopia	2011	DHS
**61.**	Gabon	2000	DHS
**62.**	Gabon	2012	DHS
**63.**	Ghana	1993	DHS
**64.**	Ghana	1998	DHS
**65.**	Ghana	2003	DHS
**66.**	Ghana	2008	DHS
**67.**	Guatemala	1995	DHS
**68.**	Guatemala	1998	DHS
**69.**	Guinea	1999	DHS
**70.**	Guinea	2005	DHS
**71.**	Guinea Bissau	2006	MICS
**72.**	Guyana	2006	MICS
**73.**	Guyana	2009	DHS
**74.**	Haiti	1994	DHS
**75.**	Haiti	2000	DHS
**76.**	Haiti	2005	DHS
**77.**	Haiti	2012	DHS
**78.**	Honduras	2005	DHS
**79.**	Honduras	2011	DHS
**80.**	India	1998	DHS
**81.**	India	2005	DHS
**82.**	Indonesia	1994	DHS
**83.**	Indonesia	1997	DHS
**84.**	Indonesia	2002	DHS
**85.**	Indonesia	2007	DHS
**86.**	Indonesia	2012	DHS
**87.**	Iraq	2006	MICS
**88.**	Jordan	1997	DHS
**89.**	Jordan	2002	DHS
**90.**	Jordan	2007	DHS

Order	Country	Year	Source
**91.**	Jordan	2012	DHS
**92.**	Kazakhstan	1995	DHS
**93.**	Kazakhstan	1999	DHS
**94.**	Kenya	1993	DHS
**95.**	Kenya	1998	DHS
**96.**	Kenya	2003	DHS
**97.**	Kenya	2008	DHS
**98.**	Kyrgyzstan	1997	DHS
**99.**	Kyrgyzstan	2005	MICS
**100.**	Lesotho	2004	DHS
**101.**	Lesotho	2009	DHS
**102.**	Liberia	2007	DHS
**103.**	Macedonia	2005	MICS
**104.**	Madagascar	1997	DHS
**105.**	Madagascar	2003	DHS
**106.**	Madagascar	2008	DHS
**107.**	Malawi	2000	DHS
**108.**	Malawi	2004	DHS
**109.**	Malawi	2010	DHS
**110.**	Maldives	2009	DHS
**111.**	Mali	1995	DHS
**112.**	Mali	2001	DHS
**113.**	Mali	2006	DHS
**114.**	Mauritania	2007	MICS
**115.**	Moldova	2005	DHS
**116.**	Mongolia	2005	MICS
**117.**	Montenegro	2005	MICS
**118.**	Morocco	2003	DHS
**119.**	Mozambique	1997	DHS
**120.**	Mozambique	2003	DHS
**121.**	Mozambique	2011	DHS
**122.**	Namibia	2000	DHS
**123.**	Namibia	2006	DHS
**124.**	Nepal	1996	DHS
**125.**	Nepal	2001	DHS
**126.**	Nepal	2006	DHS
**127.**	Nepal	2011	DHS
**128.**	Nicaragua	1997	DHS
**129.**	Nicaragua	2001	DHS
**130.**	Niger	1998	DHS
**131.**	Niger	2006	DHS
**132.**	Niger	2012	DHS
**133.**	Nigeria	1999	DHS
**134.**	Nigeria	2003	DHS
**135.**	Nigeria	2007	MICS
**136.**	Nigeria	2008	DHS
**137.**	Pakistan	2006	DHS
**138.**	Pakistan	2012	DHS
**139.**	Peru	1996	DHS
**140.**	Peru	2000	DHS
**141.**	Peru	2004	DHS
**142.**	Peru	2005	DHS
**143.**	Peru	2006	DHS
**144.**	Peru	2007	DHS

Order	Country	Year	Source
**145.**	Peru	2008	DHS
**146.**	Peru	2009	DHS
**147.**	Peru	2010	DHS
**148.**	Peru	2011	DHS
**149.**	Peru	2012	DHS
**150.**	Philippines	1993	DHS
**151.**	Philippines	1998	DHS
**152.**	Philippines	2003	DHS
**153.**	Philippines	2008	DHS
**154.**	Rwanda	2000	DHS
**155.**	Rwanda	2005	DHS
**156.**	Rwanda	2010	DHS
**157.**	Sao Tome and Principe	2008	DHS
**158.**	Senegal	1997	DHS
**159.**	Senegal	2005	DHS
**160.**	Senegal	2010	DHS
**161.**	Serbia	2005	MICS
**162.**	Sierra Leone	2008	DHS
**163.**	South Africa	1998	DHS
**164.**	Suriname	2006	MICS
**165.**	Swaziland	2006	DHS
**166.**	Syria	2006	MICS
**167.**	Tajikistan	2005	MICS
**168.**	Tajikistan	2012	DHS
**169.**	Tanzania	1996	DHS
**170.**	Tanzania	1999	DHS
**171.**	Tanzania	2004	DHS
**172.**	Tanzania	2010	DHS
**173.**	Timor Leste	2009	DHS
**174.**	Togo	1998	DHS
**175.**	Togo	2006	MICS
**176.**	Trinidad and Tobago	2006	MICS
**177.**	Turkey	1993	DHS
**178.**	Turkey	1998	DHS
**179.**	Turkey	2003	DHS
**180.**	Uganda	1995	DHS
**181.**	Uganda	2000	DHS
**182.**	Uganda	2006	DHS
**183.**	Uganda	2011	DHS
**184.**	Ukraine	2005	MICS
**185.**	Ukraine	2007	DHS
**186.**	Uzbekistan	1996	DHS
**187.**	Uzbekistan	2006	MICS
**188.**	Vietnam	1997	DHS
**189.**	Vietnam	2002	DHS
**190.**	Yemen	2006	MICS
**191.**	Zambia	1996	DHS
**192.**	Zambia	2001	DHS
**193.**	Zambia	2007	DHS
**194.**	Zimbabwe	1994	DHS
**195.**	Zimbabwe	1999	DHS
**196.**	Zimbabwe	2005	DHS
**197.**	Zimbabwe	2010	DHS

Comparison of models for the extreme wealth quintiles

We found that wealth quintiles are associated with FPC, and for any given level of CPR, wealthier women have a higher FPC (Figure S1). We considered, though, that the differences are small enough not to be taken into account in our predictive model.

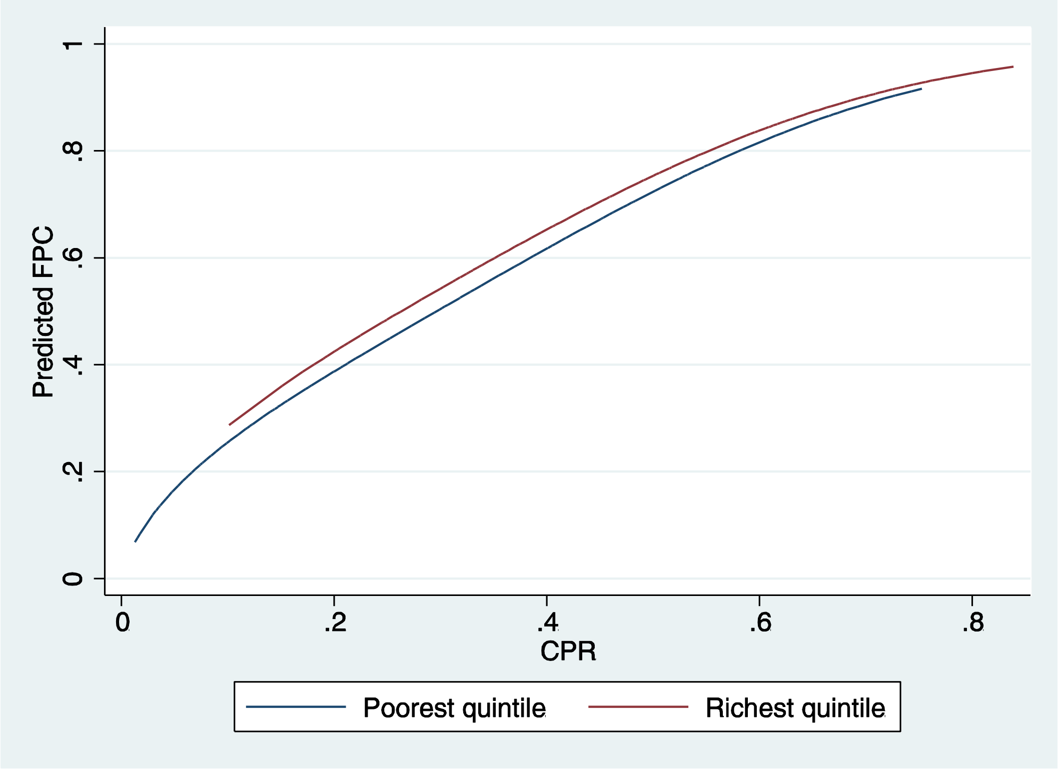

S1 Figure – Predicted values of Family Planning Coverage (FPC) for the richest (Q5) and poorest (Q1) wealth quintiles

Residual diagnostics of the predictive model

In order to check the quality of the fit obtained with our country level predictive model, we assessed the behaviour of the residuals. In Figure S2 we can see no evidence of unexplained trend, and no suggestion of increasing or decreasing variability along the predictor range of variation. In Figure S3, the normal plot suggests the residuals are very close to a normal distribution.

```
. fp <cpmt_r> : reg fpslogit <cpmt_r>, cluster(country)
(fitting 44 models)
(....10%....20%....30%....40%....50%....60%....70%....80%....90%....100%)

Linear regression                                      Number of obs =      197
                                                       F(  2,     81) =   906.31
                                                       Prob > F      =   0.0000
                                                       R-squared     =   0.9469
                                                       Root MSE      =   .25535

                              (Std. Err. adjusted for 82 clusters in country)
------------------------------------------------------------------------------
             |               Robust
    fpslogit |      Coef.   Std. Err.      t    P>|t|     [95% Conf. Interval]
-------------+----------------------------------------------------------------
    cpmt_r_1 |   .6779366   .0626886    10.81   0.000      .553206    .8026672
    cpmt_r_2 |   3.567283   .2119167    16.83   0.000     3.145635    3.988931
       _cons |   .6100581   .1141839     5.34   0.000     .3828678    .8372483
------------------------------------------------------------------------------
```

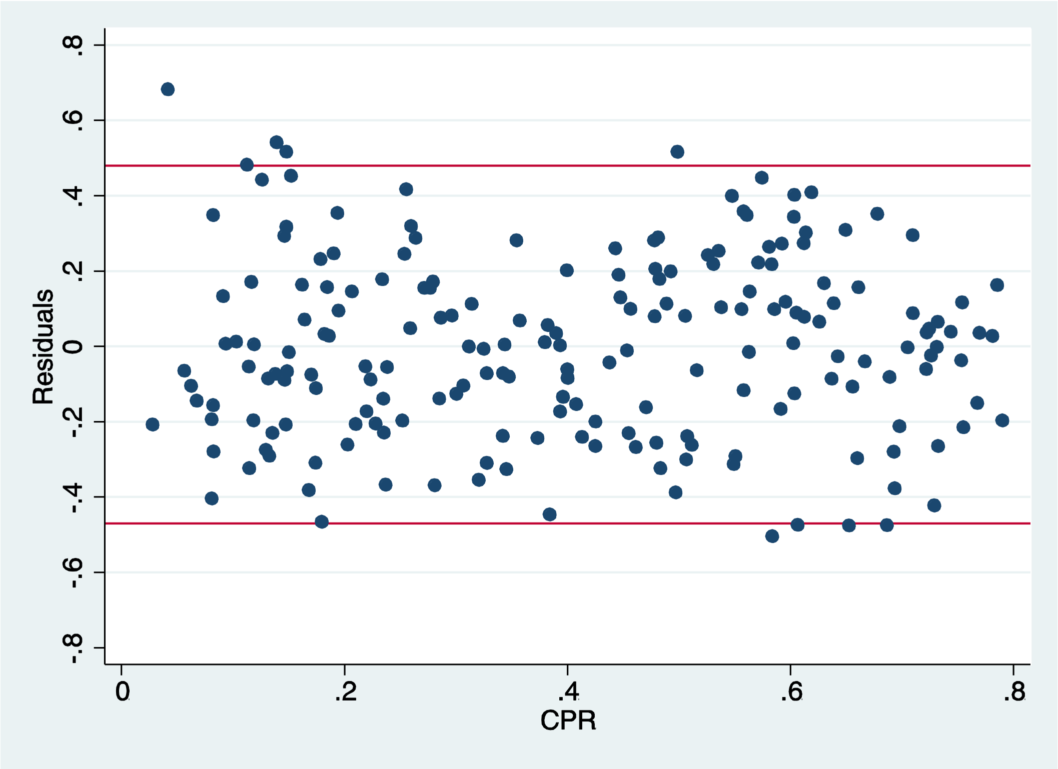

S2 Figure – Residuals vs predictor plot with lines enclosing 95% of residuals.

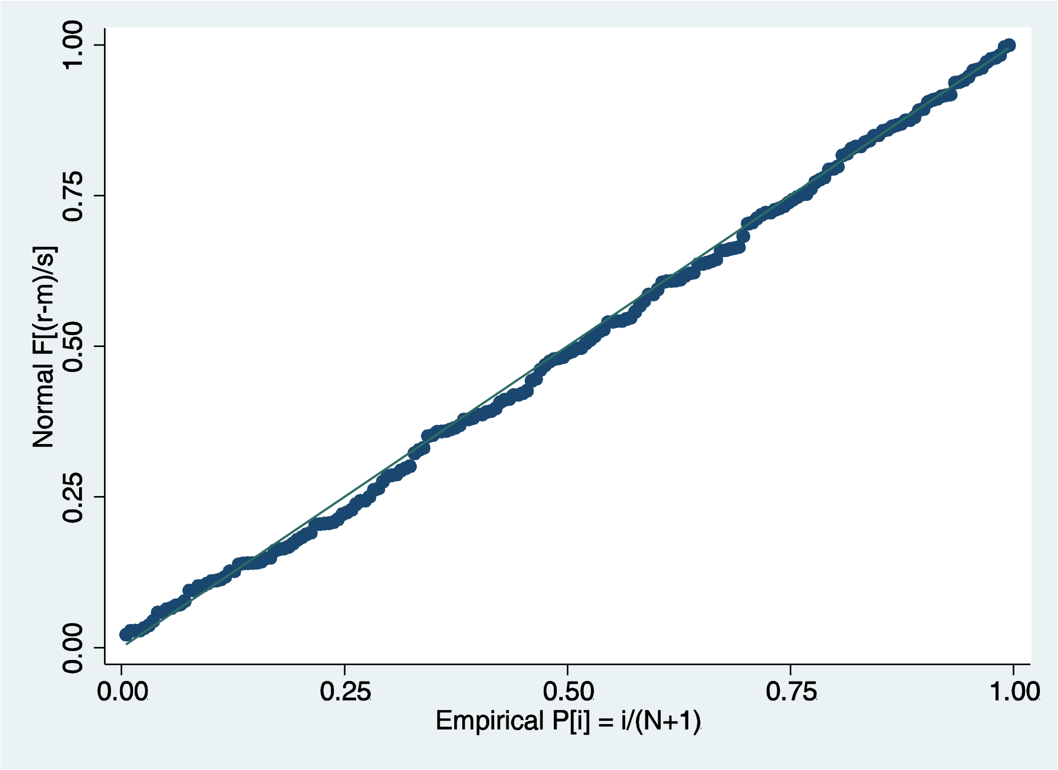

S3 Figure – Residuals normal probability plot.

Concordance between predicted and observed values of FPC

We assessed the concordance in order to have an idea of the precision for the individual prediction that is determined by the precision of the regression line plus the root of the mean squared error of the residual. In our case, the individual predictions vary approximately ±9 percent points around the true value.

```
Concordance correlation coefficient (Lin, 1989, 2000):

 rho_c    SE(rho_c)    Obs    [   95% CI   ]      P         CI type
---------------------------------------------------------------
 0.976      0.003      197     0.969  0.982     0.000   asymptotic
                               0.968  0.981     0.000  z-transform

Pearson's r =  0.976  Pr(r = 0) = 0.000  C_b = rho_c/r =  1.000
Reduced major axis:   Slope =     0.980   Intercept =     0.013

Difference = pfp1inv - fps_r

        Difference                       95% Limits Of Agreement
   Average     Std Dev.                   (Bland & Altman, 1986)
---------------------------------------------------------------
     0.001       0.048                     -0.093       0.096

Correlation between difference and mean = -0.090

Bradley-Blackwood F = 0.857 (P = 0.42613)
```

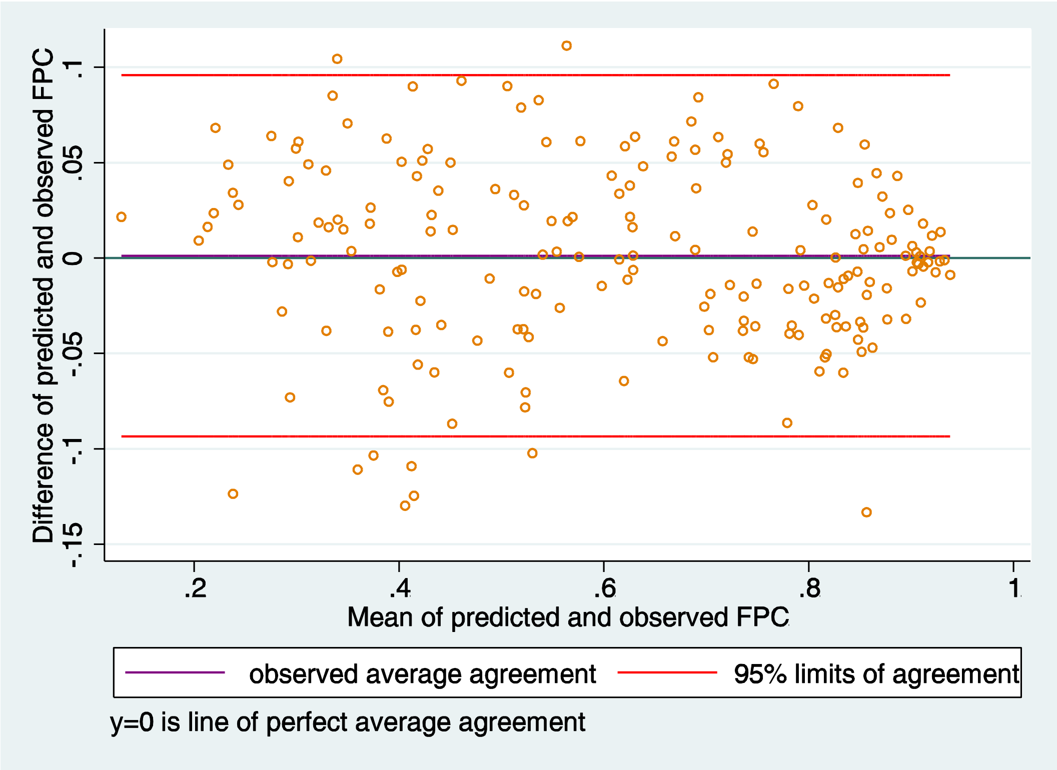

S4 Figure – Limits of agreement between predicted and observed values of family planning coverage.
